# Evaluation of exposure to contaminated drinking water and specific birth defects and childhood cancers at Marine Corps Base Camp Lejeune, North Carolina: a case–control study

**DOI:** 10.1186/1476-069X-12-104

**Published:** 2013-12-04

**Authors:** Perri Zeitz Ruckart, Frank J Bove, Morris Maslia

**Affiliations:** 1 Division of Toxicology and Human Health Sciences, Agency for Toxic Substances and Disease Registry, 4770 Buford Highway, MS F-58, Atlanta, GA 30341, USA; 2 Division of Community Health Investigations, Agency for Toxic Substances and Disease Registry, 4770 Buford Highway, MS F-59, Atlanta, GA 30341, USA

**Keywords:** Neural tube defects, Childhood cancers, Environmental epidemiology, Trichloroethylene, Water

## Abstract

**Background:**

Drinking water supplies at Marine Corps Base Camp Lejeune were contaminated with trichloroethylene, tetrachloroethylene, benzene, vinyl chloride and trans-1,2-dichloroethylene during 1968 through 1985.

**Methods:**

We conducted a case control study to determine if children born during 1968–1985 to mothers with residential exposure to contaminated drinking water at Camp Lejeune during pregnancy were more likely to have childhood hematopoietic cancers, neural tube defects (NTDs), or oral clefts. For cancers, exposures during the first year of life were also evaluated. Cases and controls were identified through a survey of parents residing on base during pregnancy and confirmed by medical records. Controls were randomly sampled from surveyed participants who had a live birth without a major birth defect or childhood cancer. Groundwater contaminant fate and transport and distribution system models provided estimates of monthly levels of drinking water contaminants at mothers’ residences. Magnitude of odds ratios (ORs) was used to assess associations. Confidence intervals (CIs) were used to indicate precision of ORs. We evaluated parental characteristics and pregnancy history to assess potential confounding.

**Results:**

Confounding was negligible so unadjusted results were presented. For NTDs and average 1^st^ trimester exposures, ORs for any benzene exposure and for trichloroethylene above 5 parts per billion were 4.1 (95% CI: 1.4-12.0) and 2.4 (95% CI: 0.6-9.6), respectively. For trichloroethylene, a monotonic exposure response relationship was observed. For childhood cancers and average 1^st^ trimester exposures, ORs for any tetrachloroethylene exposure and any vinyl chloride exposure were 1.6 (95% CI: 0.5-4.8), and 1.6 (95% CI: 0.5-4.7), respectively. The study found no evidence suggesting any other associations between outcomes and exposures.

**Conclusion:**

Although CIs were wide, ORs suggested associations between drinking water contaminants and NTDs. ORs suggested weaker associations with childhood hematopoietic cancers.

## Background

The United States Marine Corps (USMC) Base at Camp Lejeune, North Carolina began operations during the early 1940s. During the base’s 1980–85 sampling program, volatile organic compounds (VOCs) were detected in some wells in two of the base’s water distribution systems (Hadnot Point [HP] and Tarawa Terrace [TT]). Supply wells of a third water distribution system, Holcomb Boulevard (HB) were not contaminated during this sampling period.

The primary contaminant detected in the TT distribution system was tetrachloroethylene (PCE) at a maximum of 215 parts per billion (ppb). The source of the contamination was solvent waste disposal from an off-base dry cleaner [1]. The primary contaminant in the HP distribution system was trichloroethylene (TCE). The maximum level of TCE detected in the system was 1,400 ppb. Vinyl chloride and trans-1,2-dichloroethylene (DCE) were present in the distribution system due to degradation of TCE. Other major contaminants in the HP system included PCE and benzene [2]. The contaminants in the HP system resulted from leaking underground storage tanks, industrial area spills, and waste disposal sites. Both Camp Lejeune and the off-base dry cleaner are Superfund sites [3,4].

Water from contaminated and uncontaminated wells was mixed at the treatment plants before delivery to residences. Contamination levels in the drinking water distribution system varied depending on the wells being used. The most highly contaminated wells in the HP and TT systems were shut down by February 1985.

The HP, TT, and HB systems began operations during 1942, January 1952, and June 1972, respectively. Prior to June 1972, the HB service area was supplied by the HP system. In June 1972, the HB treatment plant began operations and provided drinking water to a service area previously supplied by the HP system. The HB system was supplied by wells that were uncontaminated. However, during dry weather conditions in the spring/summer months, water from the HP system supplemented the HB system. In addition, the HP system supplied water to the HB system during January 27-February 7, 1985 when the HB system was shut down for repairs. No organic solvent contamination was detected in drinking water from other on-base treatment plants.

TCE, benzene, and vinyl chloride are classified as human carcinogens [5-7]. PCE is classified as a “likely human carcinogen” [8]. The carcinogenicity of DCE is not currently classified.

Several studies have examined associations between birth defects and childhood cancers among children born to female workers exposed to solvents [9-16]. Most of these studies based exposures on job titles and did not evaluate specific solvents. Only a few studies have evaluated associations between maternal exposure to these contaminants in drinking water and birth defects and childhood cancers [17-24].

The purpose of this study is to determine if maternal exposures and exposures during the first year of life to contaminants in drinking water at Camp Lejeune increased the risk of neural tube defects (NTDs), oral clefts, and childhood hematopoietic cancers. This study received approval from the Centers for Disease Control and Prevention’s (CDC) Institutional Review Board Protocol number 4212.

## Methods

Based on the scientific literature, we initially focused on the following childhood cancers and birth defects: NTDs consisting of spina bifida and anencephaly, oral clefts consisting of cleft lip and cleft palate, conotruncal heart defects, choanal atresia, and childhood hematopoietic cancers consisting of childhood leukemia and childhood non-Hodgkin’s lymphoma (NHL).

### Study population

Since computerized birth certificates in North Carolina became available in 1968 and the contaminated wells on base were shut down in 1985, we included live births occurring between 1968 and 1985 to mothers who resided on base any time during their pregnancy. Birth defects and cancer registries were nonexistent during this time period. Therefore, we used birth certificate data to identify 12,493 children born between 1968 and 1985 to mothers who lived at Camp Lejeune at the time of delivery. A media campaign and referral process (“referral process”) were used to obtain information on an estimated additional 4,000 mothers who resided at Camp Lejeune at any time during her pregnancy, but who delivered after leaving Camp Lejeune. The media campaign, conducted by the USMC, urged Marines, Sailors and their families to contact the study helpline if they conceived a child while living at Camp Lejeune between 1968 and 1985. The referral process consisted of obtaining identifying information (name, address, phone number) for potentially eligible study participants from previously identified study participants. Names of personnel identified through referral or by the media campaign were cross-referenced with military records.

From September 1999 through January 2002, the Agency for Toxic Substances and Disease Registry (ATSDR) conducted a telephone survey and interviewed the parents of 12,598 children. Of these, 10,044 were identified from birth certificate data and 2,554 births were identified from the referral process, but we did not obtain their birth certificates. The participation rate was 76%. During the telephone survey, parents were asked if their child had a birth defect or developed a childhood cancer. In an attempt to capture all potential conditions of interest, we were very liberal in what was included in the reported categories. No cases of choanal atresia were reported in the survey. Survey participants reported less than 1/3 of the expected number of cases of conotruncal heart defects (approximately 8/10,000 live births during 1968–1985 based on surveillance data from the CDC’s Metropolitan Atlanta Congenital Defects Program, unpublished data). Due to the small number of conotruncal heart defects reported, we focused on NTDs (spina bifida and anencephaly), oral clefts (cleft lip and cleft palate), and childhood hematopoietic cancers (leukemia and NHL) diagnosed before 20 years of age.

Survey participants reported 106 cases: 35 NTDs, 42 oral clefts, and 29 childhood hematopoietic cancers. Extensive efforts were made to confirm self-reported cases by obtaining vital records information and medical records from providers or the National Personnel Records Center. In addition, for reported cases of spina bifida and oral clefts, we offered to pay for medical visits to obtain confirmation by the current medical provider. We were able to confirm 15 NTDs, 24 oral clefts, and 13 cancers. We were unable to obtain medical confirmation for 6 reported cases, 7 were ineligible, 8 refused to provide medical records, and 33 were confirmed not to have the reported condition (for example, child had another facial deformity instead of an oral cleft).

Survey participants with a live birth occurring between 1968 and 1985 who had children without a birth defect or childhood cancer were randomly selected as controls. We attempted to enroll approximately ten times as many controls as cases, using one control group for all of the cases.

### Data collection

During the telephone survey, we collected information on demographics; mother’s residential history one year before and after birth of the child; maternal water usage; mother’s medical history during pregnancy; family history of birth defects; maternal smoking, alcohol use, and occupation; and father’s lifestyle habits and occupational history. The mother and father were interviewed if available. If the mother was unavailable, we administered a shortened questionnaire to the father focusing mainly on residential history and paternal-related questions.

### Exposure assessment

Limited historical, contaminant-specific data were available, therefore ATSDR conducted a historical reconstruction of contaminant levels in the drinking water using groundwater fate and transport and water-distribution system models. Modeling provided monthly average estimates of the concentrations of the contaminants in drinking water delivered to residences. Sensitivity analyses were conducted on the calibrated water models and their resulting estimates. All information pertaining to the historical reconstruction was published in peer reviewed reports [1,2].

We used residential information collected in the interview, base family housing records, and water modeling results to assign exposures. Each month of residence was linked to estimated levels of contaminants in drinking water serving that location.

### Data analysis

We used unconditional logistic regression in SAS 9.3 to compare exposure odds of verified cases of birth defects and childhood cancers with controls. NTDs, oral clefts, and childhood hematopoietic cancers were analyzed separately. Potential risk factors were evaluated to determine any associations with outcomes. For the adjusted models, potential risk factors with odds ratios (ORs) that differed from the null value in the expected direction were included. Because of small numbers, one risk factor at a time was included in a model with the exposure variable.

Unadjusted and adjusted ORs and their 95% confidence intervals (CIs) were calculated. Adjusted models were compared to unadjusted models that only included cases and controls with complete data for the risk factor(s). Adjusted results were presented if they differed from unadjusted results by > 20%. Unless specified, unadjusted results were presented. We used two criteria to assess associations: magnitude of the OR and the exposure-response relationship. If an exposure-response relationship could be evaluated, emphasis was given to monotonic trends in the categorical exposure variables. A monotonic trend occurs when every change in the OR with increasing category of exposure is in the same direction, although the trend could have flat segments but never reverse direction [25]. Where an exposure-response relationship could not be evaluated because of small cell size, we emphasized ORs ≥1.5. Confidence intervals were used to indicate precision of ORs [26-28]. We included p-values in tables for information purposes only. We did not use statistical significance testing to interpret findings [25,27,28].

Each contaminant was evaluated separately. Analyses focused on average monthly concentration levels during specific time periods of interest for each outcome. Exposure variables were categorized such that the reference group did not have residential exposure to the contaminant under evaluation (“unexposed”). In one categorization, we divided the exposed group by the 50^th^ percentile level among controls. A second categorization divided the exposed group into two levels, below and above the EPA Maximum Contaminant Levels (MCL) for that contaminant. The current MCLs for TCE, PCE, and benzene are 5 ppb; the current MCLs for vinyl chloride and DCE are 2 ppb and 100 ppb, respectively [29]. Finally, we compared exposed versus unexposed. We excluded categorizations where there were <2 exposed cases in a cell.

Birth certificate data on gestational age at birth or last menstrual period were unavailable for some cases and controls. Therefore, date of conception (DOC) was estimated using birth date and assuming everyone was a term birth (39 weeks). For birth defects, relevant exposure windows are the 4^th^ week of gestation for NTDs and during the 6^th^-9^th^ week of gestation for oral clefts [30,31]. To ensure that we captured relevant exposure windows, we evaluated from two months prior to the estimated DOC through the first two months of gestation for NTDs. For oral clefts, exposures occurring from one month prior to the DOC through the first three months of gestation were evaluated. For childhood cancers, we evaluated each trimester, the entire pregnancy, and the first year of life.

Secondary analyses were conducted using an unexposed group consisting of those without residential exposure to any of the drinking water contaminants. We also evaluated water consumption habits. Additionally, we evaluated other exposure groupings (maximum monthly exposure, cumulative monthly exposure for cancers, and including exposure to < 1 ppb in the unexposed group). Separate analyses were conducted for cleft lip [with or without cleft palate], cleft palate, and childhood leukemia. We could not evaluate NHL separately because there were only 2 cases. Several sensitivity analyses were conducted to evaluate selection bias. We included unverified cases and recalculated ORs to determine if this changed the results. Since births identified through the referral process might constitute a biased sample, we limited analyses to cases and controls for whom we had birth certificate data. We also evaluated whether refining the exposure window using gestational age information altered results for NTD and oral clefts by restricting analyses to those births for whom we had birth certificate data. Birth certificate data, including gestational age, were available for 444 (84.4%) controls, 11 (73.3%) NTDs, 14 (58.3%) oral clefts, and 5 (38.5%) childhood cancers. Additionally, to detect potential uncontrolled confounding or other sources of bias, we evaluated 3^rd^ trimester exposures for NTDs oral clefts (non-relevant exposure windows for these birth defects based on when these organ systems are forming and susceptible to teratogens) [32]. We could not conduct the same analysis for childhood cancers because the relevant exposure window is not as well defined.

## Results and discussion

Parents of 51 (98.1%) case-children were interviewed (Table 1). Both mothers and fathers were interviewed for 43 (84.3%) cases, only the mother was interviewed for 6 (11.7%) cases, and only the father was interviewed for two (3.9%) cases. Neither parent of one (1.9%) case (a cleft palate) could be contacted. Efforts were made to contact the parents of 651 eligible control-children. Parents of 103 (15.8%) control-children refused to participate or could not be contacted. One or both parents representing 548 (84.2%) control-children were interviewed. Upon further investigation, 22 children (4.0%) were excluded as controls: 14 mothers had not lived on base at any time during the pregnancy, 6 parents were interviewed about the wrong child, and residential history during pregnancy was unavailable for two mothers. Therefore, 526 control-children were retained for analysis. Of these, the mother and father were interviewed for 348 (66.2%) controls, only the mother was interviewed for 96 (18.3%) controls, and only the father was interviewed for 82 (15.6%) controls.

**Table 1:** Frequency of outcomes of specific birth defects and childhood cancers, Camp Lejeune, 1968–1985

**Outcome**	**Total (includes cases that could not be verified)**	**Total verified**		**Parent interviewed**	
		**Frequency**	**%**	**Frequency**	**% Interviewed**
**Neural tube defects**	17	15	--	15	100.0
- anencephaly	7	6	40.0	6	100.0
- spina bifida	10	9	60.0	9	100.0
**Oral cleft defect**	27	24	--	23	95.8
- cleft palate	12	11	45.8	10	91.0
- cleft lip (with or without cleft palate)	15	13	54.2	13	100.0
**Childhood hematopoietic cancers**	14	13	--	13	100.0
- leukemia	11	11	84.6	11	100.0
- non-Hodgkin’s lymphoma	3	2	15.4	2	100.0
**Total**	58	52	--	51	98.1

Potential risk factors from parental interviews are shown in Table 2. Mother’s age was categorized as <20 or ≥20 because of small numbers of mothers over age 30. Mothers of cases reported drinking more glasses of tap water per day than mothers of controls (Table 3). Mothers of NTDs and oral clefts were similar to mothers of controls for frequency of showering, however, more mothers of cancer cases showered ≥ 14 times a week.

**Table 2:** Risk factors for specific birth defects and childhood cancers, Camp Lejeune, 1968–1985

**Potential risk factor**	Controls*	**Neural tube defects**		**Oral clefts**		Cancers**	
	**# (%)**	**# (%) OR (95%CI)**		**# (%) OR (95%CI)**		**# (%) OR (95%CI)**	
Maternal age							
<20	86 (16.4)	5 (33.3)	2.6 (0.9, 7.6)	4 (16.7)	1.0 (0.3, 3.1)	2 (15.4)	0.9 (0.2, 4.3)
≥20	438 (83.6)	10 (66.7)	1.0 (ref.)	20 (83.3)	1.0 (ref.)	11 (84.6)	1.0 (ref.)
Maternal education							
Not a college graduate	375 (72.1)	10 (66.7)	0.8 (0.3, 2.3)	19 (82.6)	1.8 (0.6, 5.5)	12 (92.3)	4.6 (0.6, 36.0)
College graduate	145 (27.9)	5 (33.3)	1.0 (ref.)	4 (17.4)	1.0 (ref.)	1 (7.7)	1.0 (ref.)
Prenatal Care†							
“Inadequate”	44 (9.2)	1 (6.7)	0.7 (0.1, 5.5)	2 (9.1)	1.0 (0.2, 4.4)	1 (8.3)	0.9 (0.1, 7.1)
“Adequate”	436 (90.8)	14 (93.3)	1.0 (ref.)	20 (90.9)	1.0 (ref.)	11 (91.7)	1.0 (ref.)
Prenatal vitamins, 1^st^ trimester							
No	64 (14.9)	2 (13.3)	0.9 (0.2, 4.0)	4 (18.2)	1.3 (0.4, 3.9)	1 (8.3)	0.5 (0.1, 4.1)
Yes	365 (85.1)	13 (86.7)	1.0 (ref.)	18 (81.8)	1.0 (ref.)	11 (91.7)	1.0 (ref.)
1^st^ pregnancy	168 (32.4)	7 (46.7)	1.8 (0.7, 5.1)	12 (52.2)	2.3 (1.0, 5.3)	1 (8.3)	0.2 (0.0, 1.5)
>1 pregnancy	351 (67.6)	8 (53.3)	1.0 (ref.)	11 (47.8)	1.0 (ref.)	11 (91.7)	1.0 (ref.)
Mother worked, 1^st^ trimester							
Yes	76 (15.3)	1 (7.7)	0.5 (0.1, 3.6)	5 (25.0)	1.9 (0.7, 5.2)	2 (15.4)	1.0 (0.2, 4.6)
No	421 (84.7)	12 (92.3)	1.0 (ref.)	15 (75.0)	1.0 (ref.)	11 (84.6)	1.0 (ref.)
Smoking, 1^st^ trimester							
Yes	130 (29.3)	1 (6.7)	0.2 (0.0, 1.3)	4 (18.2)	0.5 (0.2, 1.6)	5 (41.7)	1.7 (0.5, 5.5)
No	314 (70.7)	14 (93.3)	1.0 (ref.)	18 (81.8)	1.0 (ref.)	7 (58.3)	1.0 (ref.)
Alcohol, 1^st^ trimester							
Yes	96 (21.7)	3 (21.4)	1.0 (0.3, 3.6)	7 (31.8)	1.7 (0.7, 4.3)	1 (8.3)	0.3 (0.0, 2.6)
No	347 (78.3)	11 (78.6)	1.0 (ref.)	15 (68.2)	1.0 (ref.)	11 (91.7)	1.0 (ref.)
Fevers, 1^st^ trimester							
Yes	45 (10.7)	1 (6.7)	0.6 (0.1, 4.6)	3 (13.6)	1.3 (0.4, 4.6)	2 (18.2)	1.9 (0.4, 8.9)
No	376 (89.3)	14 (93.3)	1.0 (ref.)	19 (86.4)	1.0 (ref.)	9 (81.8)	1.0 (ref.)
Passive smoke, 1^st^ trimester							
Yes	203 (45.9)	5 (33.3)	0.6 (0.2, 1.8)	6 (27.3)	0.4 (0.2, 1.2)	7 (58.3)	1.7 (0.5, 5.3)
No	239 (54.1)	10 (66.7)	1.0 (ref.)	16 (72.7)	1.0 (ref.)	5 (41.7)	1.0 (ref.)
Child’s sex							
Male	274 (52.1)	6 (40.0)	0.6 (0.2, 1.8)	9 (37.5)	0.6 (0.2, 1.3)	8 (61.5)	1.5 (0.5, 4.6)
Female	252 (47.9)	9 (60.0)	1.0 (ref.)	15 (67.5)	1.0 (ref.)	5 (38.5)	1.0 (ref.)
Child’s race							
Non-white	131 (25.0)	1 (6.7)	0.2 (0.0,1.6)	6 (25.0)	1.0 (0.4, 2.6)	5 (38.5)	1.9 (0.6-5.8)
“White”	393 (75.0)	14 (93.3)	1.0 (ref.)	18 (75.0)	1.0 (ref.)	8 (61.5)	1.0 (ref.)
Child’s sibling has birth defect							
Yes	45 (11.0)	2 (14.3)	1.3 (0.3, 6.2)	6 (27.3)	3.0 (1.1, 8.2)	3 (27.3)	3.0 (0.8, 11.8)
No	364 (89.0)	12 (85.7)	1.0 (ref.)	16 (72.7)	1.0 (ref.)	8 (72.7)	1.0 (ref.)
Dad smoked † †							
Yes	273 (52.6)	6 (40.0)	0.6 (0.2, 1.7)	8 (36.4)	0.5 (0.2, 1.3)	9 (69.2)	2.0 (0.6, 6.7)
No	246 (47.4)	9 (60.0)	1.0 (ref.)	14 (63.6)	1.0 (ref.)	4 (30.8)	1.0 (ref.)
Dad possibly exposed to Agent Orange							
Yes	158 (31.2)	3 (20.0)	0.6 (0.2, 2.0)	6 (26.1)	0.8 (0.3, 2.0)	5 (38.5)	1.4 (0.4, 4.3)
No	348 (68.8)	12 (80.0)	1.0 (ref.)	17 (73.9)	1.0 (ref.)	8 (61.5)	1.0 (ref.)
Dad occupationally exposed to solvents §							
Yes	167 (32.5)	6 (40.0)	1.4 (0.5-3.9)	6 (26.1)	0.7 (0.3-1.9)	4 (30.8)	0.9 (0.3-3.0)
No	347 (67.5)	9 (60.0)	1.0 (ref.)	17 (73.9)	1.0 (ref.)	9 (69.2)	1.0 (ref.)

*one control series for all case groups.

** childhood leukemia and childhood non-Hodgkin’s lymphoma.

† “Adequate”: prenatal care began during 1^st^ trimester; “Inadequate”: prenatal care began later in pregnancy or no care was received.

† † During the three months before conception.

§ During the six months before conception.

**Table 3:** Mother’s water consumption habits during the first trimester of pregnancy, Camp Lejeune, 1968–1985

**Maternal water usage**	**Controls**		**Neural tube defects**		**Oral cleft defects**		Cancers^*^	
	**#**	**%**	**#**	**%**	**#**	**%**	**#**	**%**
Daily average glasses of tap water								
≤5	256	49.7	4	26.7	9	39.1	2	15.4
>5	259	50.3	11	73.3	14	60.9	11	84.6
Frequency mother showered or bathed								
≤7/week	390	74.1	11	73.3	17	70.8	9	69.2
8 – 13/week	43	8.2	1	6.7	3	12.5	0	0.0
≥14/week	93	17.7	3	20.0	4	16.7	4	30.8

*childhood leukemia and childhood non-Hodgkin’s lymphoma.

For NTDs and average 1^st^ trimester exposures, the OR for TCE above the MCL was 2.4 (95% CI: 0.6-9.6), and we observed a monotonic exposure response relationship for exposures categorized using the MCL. The OR for any benzene exposure was 4.1 (95% CI: 1.4-12.0), but we could not evaluate exposure response relationships because there were <2 cases in the high exposure category (Table 4). For oral clefts and the contaminants evaluated, all ORs were ≤ 1.0 (Table 5). For childhood cancers and average 1^st^ trimester exposures, the OR for any PCE exposure was 1.6 (95% CI: 0.5-4.8), the OR for any vinyl chloride exposure was 1.6 (95% CI: 0.5-4.7), and the OR for any DCE exposure was 1.5 (95% CI: 0.5-4.7) however, risk did not increase with increasing categories of exposure (Table 6).

**Table 4:** Neural tube defects and average VOC exposure*, first trimester, Camp Lejeune, 1968–1985

	**Controls**	**Neural tube defects** **Unadjusted**		**p-value**
	**# (%)**	**# (%) OR (95%CI)**		
**PCE**				
Unexposed	330 (62.7)	10 (66.7)	1.0 (ref.)	
Below MCL (>0- ≤ 5 ppb)	27 (5.1)	3 (20.0)	3.7 (1.0-14.1)	0.06
Above MCL (> 5 ppb)	169 (32.1)	2 (13.3)	0.4 (0.1- 1.8)	0.23
Unexposed	330 (62.7)	10 (66.7)	1.0 (ref.)	
Exposed	196 (37.2)	5 (33.3)	0.8 (0.3-2.5)	0.76
**TCE**				
Unexposed	287 (54.6)	7 (46.7)	1.0 (ref.)	
Low (>0- ≤ 2 ppb)	114 (21.7)	4 (26.7)	1.4 (0.4-5.0)	0.57
High (> 2 ppb)	125 (23.8)	4 (26.7)	1.3 (0.4-4.6)	0.67
Unexposed	287 (54.6)	7 (46.7)	1.0 (ref.)	
Below MCL (>0- ≤ 5 ppb)	188 (35.7)	5 (33.3)	1.1 (0.3-3.5)	0.88
Above MCL (>5 ppb)	51 (9.7)	3 (20.0)	2.4 (0.6-9.6)	0.21
Unexposed	287 (54.6)	7 (46.7)	1.0 (ref.)	
Exposed	239 (45.4)	8 (53.3)	1.4 (0.5-3.8)	0.55
**Benzene**				
Unexposed	453 (86.1)	9 (60.0)	1.0 (ref.)	
Exposed	73 (13.9)	6 (40.0)	4.1 (1.4-12.0)	0.01
**Vinyl Chloride**				
Unexposed	329 (62.5)	9 (60.0)	1.0 (ref.)	
Exposed	197 (37.5)	6 (40.0)	1.1 (0.4-3.2)	0.84
**DCE**				
Unexposed	328 (62.4)	9 (60.0)	1.0 (ref.)	
Exposed	198 (37.6)	6 (40.0)	1.1 (0.4-3.1)	0.85

*when possible, we divided the exposed group by the 50^th^ percentile level among controls (low and high); we excluded categorizations where there were <2 exposed cases in a cell.

**Table 5:** Oral cleft defects and average VOC exposure*, first trimester, Camp Lejeune, 1968–1985

	**Controls**	**Oral clefts** **Unadjusted**		**p-value**
	**# (%)**	**# (%) OR (95%CI)**		
**PCE**				
Unexposed	304 (57.8)	17 (70.8)	1.0 (ref.)	
Low (>0- < 44 ppb)	111 (21.1)	4 (16.7)	0.6 (0.2-2.0)	0.43
High (≥ 44 ppb)	111 (21.1)	3 (12.5)	0.5 (0.1-1.7)	0.25
Unexposed	304 (57.8)	17 (70.8)	1.0 (ref.)	
Below MCL (>0- ≤ 5 ppb)	37 (7.0)	2 (8.3)	1.0 (0.2-4.4)	0.96
Above MCL (> 5 ppb)	185 (35.2)	5 (20.8)	0.5 (0.2-1.3)	0.16
Unexposed	304 (57.8)	17 (70.8)	1.0 (ref.)	
Exposed	222 (42.2)	7 (29.2)	0.6 (0.2-1.4)	0.21
**TCE**				
Unexposed	253 (48.1)	15 (62.5)	1.0 (ref.)	
Low (>0- ≤ 2 ppb)	130 (24.7)	4 (16.7)	0.5 (0.2-1.6)	0.25
High (> 2 ppb)	143 (27.2)	5 (20.8)	0.6 (0.2-1.7)	0.32
Unexposed	253 (48.1)	15 (62.5)	1.0 (ref.)	
Below MCL (>0- ≤ 5 ppb)	212 (40.3)	6 (25.0)	0.5 (0.2-1.3)	0.13
Above MCL (>5 ppb)	61 (11.6)	3 (12.5)	0.8 (0.2-3.0)	0.77
Unexposed	253 (48.1)	15 (62.5)	1.0 (ref.)	
Exposed	273 (51.9)	9 (37.5)	0.6 (0.2-1.3)	0.17
**Benzene**				
Unexposed	432	21 (87.5)	1.0 (ref.)	
Exposed	94	3 (12.5)	0.7 (0.2-2.2)	0.50
**Vinyl Chloride**				
Unexposed	301 (57.2)	17 (70.8)	1.0 (ref.)	
Low (>0- < 3 ppb)	141 (26.8)	4 (16.7)	0.5 (0.2-1.5)	0.22
High (≥ 3 ppb)	84 (16.0)	3 (12.5)	0.6 (0.2-2.2)	0.47
Unexposed	301 (57.2)	17 (70.8)	1.0 (ref.)	
Below MCL (>0- ≤ 2 ppb)	74 (14.1)	4 (16.7)	1.0 (0.3-3.0)	0.94
Above MCL (> 2 ppb)	151 (28.7)	3 (12.5)	0.4 (0.1-1.2)	0.10
Unexposed	301 (57.2)	17 (70.8)	1.0 (ref.)	
Exposed	225 (42.8)	7 (29.2)	0.6 (0.2-1.4)	0.19
**DCE**				
Unexposed	300 (57.0)	17 (70.8)	1.0 (ref.)	
Low (>0- < 5 ppb)	116 (22.1)	4 (16.7)	0.6 (0.2-1.8)	0.38
High (≥ 5 ppb)	110 (20.9)	3 (12.5)	0.5 (0.1-1.7)	0.25
Unexposed	300 (57.0)	17 (70.8)	1.0 (ref.)	
Exposed	226 (43.0)	7 (29.2)	0.5 (0.2-1.3)	0.19

*when possible, we divided the exposed group by the 50^th^ percentile level among controls (low and high); we excluded categorizations where there were <2 exposed cases in a cell.

**Table 6:** Childhood cancers* and average VOC exposure**, first trimester, Camp Lejeune, 1968–1985

	**Controls**	**Cancers** **Unadjusted**		**p-value**
	**# (%)**	**# (%) OR (95%CI)**		
**PCE**				
Unexposed	304 (57.8)	6 (46.2)	1.0 (ref.)	
Low (>0- < 44 ppb)	111 (21.1)	4 (30.8)	1.8 (0.5-6.6)	0.36
High (≥ 44 ppb)	111 (21.1)	3 (23.1)	1.4 (0.3-5.6)	0.66
Unexposed	304 (57.8)	6 (46.2)	1.0 (ref.)	
Exposed	222 (42.2)	7 (53.8)	1.6 (0.5-4.8)	0.41
**TCE**				
Unexposed	253 (48.1)	6 (46.2)	1.0 (ref.)	
Low (>0- ≤ 2 ppb)	130 (24.7)	5 (38.5)	1.6 (0.5-5.4)	0.43
High (> 2 ppb)	143 (27.2)	2 (15.4)	0.6 (0.1-3.0)	0.52
Unexposed	253 (48.1)	6 (46.2)	1.0 (ref.)	
Exposed	273 (51.9)	7 (53.8)	1.1 (0.4-3.3)	0.89
**Benzene**				
Unexposed	432	11 (84.6)	1.0 (ref.)	
Exposed	94	2 (15.4)	0.8 (0.2-3.8)	0.82
**Vinyl Chloride**				
Unexposed	301 (57.2)	6 (46.2)	1.0 (ref.)	
Low (>0- < 3 ppb)	141 (26.8)	5 (38.5)	1.8 (0.5-6.0)	0.35
High (≥ 3 ppb)	84 (16.0)	2 (15.4)	1.2 (0.2-6.0)	0.83
Unexposed	301 (57.2)	6 (46.2)	1.0 (ref.)	
Below MCL (>0- ≤ 2 ppb)	74 (14.1)	3 (23.1)	2.0 (0.5-8.3)	0.32
Above MCL (> 2 ppb)	151 (28.7)	4 (30.8)	1.3 (0.4-4.8)	0.66
Unexposed	301 (57.2)	6 (46.2)	1.0 (ref.)	
Exposed	225 (42.8)	7 (53.8)	1.6 (0.5-4.7)	0.43
**DCE**				
Unexposed	300 (57.0)	6 (46.2)	1.0 (ref.)	
Low (>0- < 5 ppb)	116 (22.1)	4 (30.8)	1.7 (0.5-6.2)	0.41
High (≥ 5 ppb)	110 (20.9)	3 (23.1)	1.4 (0.3-5.5)	0.66
Unexposed	300 (57.0)	6 (46.2)	1.0 (ref.)	
Exposed	226 (43.0)	7 (53.8)	1.5 (0.5-4.7)	0.44

*childhood leukemia and childhood non-Hodgkin’s lymphoma.

**when possible, we divided the exposed group by the 50^th^ percentile level among controls (low and high); we excluded categorizations where there were <2 exposed cases in a cell.

When adjusting for potential risk factors, a child’s sibling reportedly having a birth defect increased the OR (adjusted OR = 1.1, 95% CI: 0.2-5.4 versus unadjusted OR = 0.8, 95% CI: 0.2-3.8) for the model for childhood cancers and benzene. However, this was based on two exposed cases. An additional file shows this information [See Additional file 1]. Adjusting for other potential risk factors either did not affect the OR or made no appreciable difference.

We obtained similar results comparing average 1^st^ trimester exposures to each VOC to those without residential drinking water exposure to any VOC. An additional file shows this information [See Additional files 2 and 3]. We also categorized 1^st^ trimester exposure for each contaminant as mothers reported drinking ≤5 glasses of water per day or > 5 glasses of water per day. Comparing these groupings with the unexposed, associations were seen for NTDs and TCE (OR =2.1, 95% CI: 0.7-6.2) among those who reported drinking >5 glasses of water per day. An additional file shows this information [See Additional files 4 and 5]. However, we could not evaluate all exposures because some of the categorizations had less than two exposed cases.

Analyses using other 1^st^ trimester exposure groupings (maximum, unexposed included < 1 ppb) produced similar results as analyses with average exposure. For cancers, ORs for exposures to TCE and benzene during other time periods examined were < 1.0. We obtained similar results comparing average exposures in these time periods with a group that did not have residential drinking water exposure to any VOC. Additionally, no association was seen for cumulative exposure to each VOC from the approximate DOC through the first year of life or through the entire pregnancy (results not shown).

Analyses evaluating cleft lip (with or without cleft palate) and cleft palate separately were similar to analyses evaluating both oral cleft defects combined, except the OR for cleft palate and average 1^st^ trimester TCE exposure > MCL was elevated (cleft palate OR = 1.4, 95% CI: 0.3-7.0 versus the combined oral clefts OR = 0.8, 95% CI: 0.2-3.0), but this was based on two exposed cases. Analyses evaluating childhood leukemia separately showed similar results as analyses evaluating both cancers combined.

We had birth certificates for more controls, NTDs, and oral clefts. When births were aggregated into three six year birth intervals covering the study period, controls and NTDs were fairly evenly distributed between those for whom we did and did not have a birth certificate. When evaluating birth intervals, more oral clefts came from the referral process in the earliest time period and more childhood cancers came from the referral process in later time periods. When births were restricted to those for whom we had birth certificates, ORs were strengthened for NTDs, similar for oral clefts, and weakened for childhood cancers compared to those including all births. We obtained similar results when we included cases whom could not be confirmed as having or not having the reported condition. Restricting analyses to those births for whom we had gestational age and narrowing exposure assessment to the 1^st^ month of pregnancy for NTDs and the 2^nd^ and 3^rd^ months of pregnancy for oral clefts produced similar results as analyses of all births and average VOC exposures during the 1^st^ trimester. For analyses using a non-relevant exposure window (i.e., 3^rd^ trimester), ORs were ≤ 1.0 for NTDs and TCE and benzene and for oral clefts and benzene (results not shown).

This study is unique because it thoroughly examined associations between modeled drinking water contamination and risk of developing specific birth defects and childhood cancers. Efforts were made to achieve a complete ascertainment of all cases of NTDs, oral clefts, and childhood hematopoetic cancers. Computer modeling of drinking water systems at Camp Lejeune during 1968–1985 provided ATSDR with extensive exposure estimates [1,2]. Errors in recalling maternal residential address on base during pregnancy were minimized by cross-referencing survey responses with family housing records.

A monotonic exposure response relationship was observed for NTDs and 1^st^ trimester exposure to TCE with an OR of 2.4 when TCE was categorized using the MCL. A similar finding was observed when mothers’ self-reported water consumption during the 1^st^ trimester was considered. Our finding for TCE and NTDs is consistent with a previous study conducted elsewhere [18]. We could not evaluate exposure-response trends for benzene because of small numbers. However, an OR of 4.1 was also observed for NTDs and any 1^st^ trimester exposure to benzene. This finding is also consistent with a previous study conducted elsewhere [18].

ORs between 1.5 and 1.6 were observed for childhood hematopoietic cancers and any 1^st^ trimester exposures to PCE, vinyl chloride, and DCE. We did not observe exposure-response trends for childhood cancer. Although two drinking water studies conducted elsewhere have observed associations between PCE contaminated drinking water and childhood leukemia, PCE was not the main contaminant in either study [19,23]. We are unaware of any previous studies linking drinking water exposures to vinyl chloride or DCE and childhood hematopoietic cancers.

Exposures to the contaminants in the drinking water at Camp Lejeune did not increase the risk of oral clefts, as indicated by ORs ≤ 1.0. A few studies have also found ORs ≤ 1.0 for oral clefts and occupational solvent exposures [10,33,34]. However, other studies of occupational solvent exposures found associations with oral clefts [35]; and a study in Cape Cod found an association between exposures to PCE in drinking water and oral clefts [17].

Results obtained when we restricted analyses to births for whom we had gestational age did not appreciably differ from results obtained assuming all births occurred at term and using imprecise estimates of relevant exposure windows. Therefore, it appears that the exposure assessment used for the analyses including all births is an appropriate surrogate measure.

Mother’s age is a known risk factor for NTDs [36] and an association was observed in this analysis, however mother’s age was not a confounder in this study. Paternal smoking at time of conception, parental age, and family size have been shown to be risk factors for childhood cancer [37,38]. Paternal, maternal, and passive smoking were not confounders in this study. Parental age was not assessed because it was not independently associated with the outcome in our study, and data on family size were not available. We were unable to assess maternal occupational exposure to solvents because no mothers of cases reported working with these chemicals.

Although selection bias is possible because some participants came from the referral process, sensitivity analyses indicated that such a bias might be minimal. In particular, results of analyses restricted to those for whom we had birth certificates were similar to results obtained using all cases and controls. Lack of an association when analyzing non-relevant exposure windows for the birth defects supports the assumption that there is no potential uncontrolled confounding or selection bias that would bias the results away from the null [32].

### Limitations

The findings were based on small numbers of cases which resulted in low precision (wide confidence intervals) for the ORs. Despite extensive efforts, we were unable to confirm six reported cases. Cases were identified through a survey which is a poor method of ascertainment. Even though the survey achieved a high participation rate of almost 80% of the estimated number of pregnancies occurring at Camp Lejeune during the study period, rates of birth defects and childhood cancers among the non-participants are unknown. Interviews were conducted from 20–37 years after the births which likely contributed to errors in recall and missing data for potential risk factors and water consumption habits. Because some contaminants were correlated (e.g., TCE, DCE, and benzene) and we had small numbers of cases, it was difficult to distinguish effects of one chemical independent of the other. Additionally because of small numbers of cases, we could not evaluate more than one chemical in a model. We did not have data on gestational age at birth for all participants or mothers’ exposures to contaminated drinking water on base at locations other than their residences. Although we used a comprehensive exposure assessment, it is probable that exposure misclassification occurred which likely biased results toward the null in comparisons involving two levels and distorted exposure-response trends in comparisons involving more than two levels.

## Conclusion

ORs suggested associations between 1^st^ trimester exposure to TCE and benzene and NTDs, and we observed a monotonic exposure response relationship for TCE. ORs suggested weaker associations between 1^st^ trimester exposure to PCE, vinyl chloride, and DCE and childhood hematopoietic cancers. However, the ORs were imprecise having wide CIs. The study found no evidence suggesting any other associations between outcomes and exposures. This study modeled monthly exposures to VOCs in drinking water. Results of this study add to the scientific literature on the health effects of exposures to these chemicals in drinking water. Additionally, results of this study may be used in conjunction with results from other studies to guide future policy decisions such as regulating levels of these contaminants in drinking water. Because the research in this area is limited, additional studies may be warranted in other populations to further assess the relationship between VOCs and these outcomes when there are registries to identify cases and exposure information can be well characterized.

### Consent

Oral informed consent was obtained from the participants for the publication of this report and any accompanying images.

## Abbreviations

DCE: 1,2-dichloroethylene; ATSDR: Agency for Toxic Substances and Disease Registry; CDC: Centers for Disease Control and Prevention; CI: Confidence interval; DOC: Date of conception; HP: Hadnot point; HB: Holcomb Boulevard; MCL: Maximum contaminant level; MACDP: Metropolitan Atlanta Congenital Defects Program; NTDs: Neural tube defects; NHL: Non-Hodgkin’s lymphoma; OR: Odds ratio; ppb: Parts per billion; PHA: Public health assessment; TT: Tarawa Terrace; PCE: Tetrachloroethylene/perchloroethylene; TCE: Trichloroethylene; USMC: United States Marine Corps; VOCs: Volatile organic compounds; EPA: United States Environmental Protection Agency; μg: Micrograms.

## Competing interests

The authors declare that they have no competing interests.

## Authors’ contributions

PZR participated in the study design, data collection, analysis and interpretation of data, and drafted the manuscript. FJB participated in the study design, data collection, interpretation of data, and helped draft the manuscript. MM conducted the water modeling. All authors read and approved the final manuscript.

## Supplementary Material

Additional file 1 **a. Neural tube defects and first trimester VOC exposure, adjusted results, Camp Lejeune, 1968-1985.** b. Oral clefts and first trimester VOC exposure*, adjusted results, Camp Lejeune, 1968-1985. c. Childhood cancers and first trimester VOC exposure**, adjusted results, Camp Lejeune, 1968-1985. Click here for file

Additional file 2 Neural tube defects and first trimester VOC exposure (unexposed group had no exposure to any VOCs), Camp Lejeune, 1968-1985. Click here for file

Additional file 3 Oral clefts and childhood cancers and first trimester VOC exposure (unexposed group had no exposure to any VOCs), Camp Lejeune, 1968-1985. Click here for file

Additional file 4 Neural tube defects and first trimester VOC exposure, accounting for water consumption, Camp Lejeune, 1968-1985*. Click here for file

Additional file 5 Oral clefts and childhood cancers and first trimester VOC exposure, accounting for water consumption, Camp Lejeune, 1968-1985*. Click here for file
