# Supplementary material for: Evaluation of exposure to contaminated drinking water and specific birth defects and childhood cancers at Marine Corps Base Camp Lejeune, North Carolina: a case–control study
Source: Environ Health. 2013 Dec 4;12:104. doi: 10.1186/1476-069X-12-104 (PMC3880212; doi:10.1186/1476-069X-12-104)
Supplement: Additional file 1 — a. Neural tube defects and first trimester VOC exposure, adjusted results, Camp Lejeune, 1968-1985. b. Oral clefts and first trimester VOC exposure*, adjusted results, Camp Lejeune, 1968-1985. c. Childhood cancers and first trimester VOC exposure**, adjusted results, Camp Lejeune, 1968-1985. [file 1476-069X-12-104-S1.doc]

**Additional file 1a.** **Neural tube defects and first trimester VOC exposure, adjusted results, Camp Lejeune, 1968-1985.**

| **Adjusted risk factors** | **Controls**  **#** | **Neural Tube Defects**  **# OR (95% CI)** | |
| --- | --- | --- | --- |
| **Mother’s age (< 20 vs. ≤ 20)** |
| Unexposed | 330 | 10 | 1.0 (ref.) |
| PCE exposure | 196 | 5 | 1.0 (0.3-2.9) |
| Unexposed | 287 | 7 | 1.0 (ref.) |
| TCE exposure | 239 | 8 | 1.6 (0.5-4.5) |
| Unexposed | 453 | 9 | 1.0 (ref.) |
| Benzene exposure | 73 | 6 | 4.4 (1.5-12.8) |
| Unexposed | 329 | 9 | 1.0 (ref.) |
| VC exposure | 197 | 6 | 1.3 (0.4-3.8) |
| Unexposed | 328 | 9 | 1.0 (ref.) |
| DCE exposure | 198 | 6 | 1.3 (0.4-3.8) |

| **Previous pregnancy** |  |  | |
| --- | --- | --- | --- |
| Unexposed | 330 | 10 | 1.0 (ref.) |
| PCE exposure | 196 | 5 | 0.8 (0.3-2.5) |
| Unexposed | 287 | 7 | 1.0 (ref.) |
| TCE exposure | 239 | 8 | 1.4 (0.5-3.9) |
| Unexposed | 453 | 9 | 1.0 (ref.) |
| Benzene exposure | 73 | 6 | 4.2 (1.4-12.0) |
| Unexposed | 329 | 9 | 1.0 (ref.) |
| VC exposure | 197 | 6 | 1.1 (0.4-3.2) |
| Unexposed | 328 | 9 | 1.0 (ref.) |
| DCE exposure | 198 | 6 | 1.1 (0.4-3.2) |
| **Sex of child** |  |  | |
| Unexposed | 330 | 10 | 1.0 (ref.) |
| PCE exposure | 196 | 5 | 0.8 (0.3-2.4) |
| Unexposed | 287 | 7 | 1.0 (ref.) |
| TCE exposure | 239 | 8 | 1.3 (0.5-3.8) |
| Unexposed | 453 | 9 | 1.0 (ref.) |
| Benzene exposure | 73 | 6 | 4.1 (1.4-11.9) |
| Unexposed | 329 | 9 | 1.0 (ref.) |
| VC exposure | 197 | 6 | 1.1 (0.4-3.1) |
| Unexposed | 328 | 9 | 1.0 (ref.) |
| DCE exposure | 198 | 6 | 1.1 (0.4-3.0) |
| **Child’s sibling with a birth defect** |  |  | |
| Unexposed | 330 | 10 | 1.0 (ref.) |
| PCE exposure | 196 | 5 | 0.9 (0.3-2.8) |
| Unexposed | 287 | 7 | 1.0 (ref.) |
| TCE exposure | 239 | 8 | 1.2 (0.4-3.5) |
| Unexposed | 453 | 9 | 1.0 (ref.) |
| Benzene exposure | 73 | 6 | 4.8 (1.6-14.5) |
| Unexposed | 329 | 9 | 1.0 (ref.) |
| VC exposure | 197 | 6 | 0.9 (0.3-2.8) |
| Unexposed | 328 | 9 | 1.0 (ref.) |
| DCE exposure | 198 | 6 | 0.9 (0.3-2.8) |
| **Father occupationally exposed to solvents*** |  |  | |
| Unexposed | 330 | 10 | 1.0 (ref.) |
| PCE exposure | 196 | 5 | 0.8 (0.3-2.5) |
| Unexposed | 287 | 7 | 1.0 (ref.) |
| TCE exposure | 239 | 8 | 1.4 (0.5-3.9) |
| Unexposed | 453 | 9 | 1.0 (ref.) |
| Benzene exposure | 73 | 6 | 4.2 (1.4-12.1) |
| Unexposed | 329 | 9 | 1.0 (ref.) |
| VC exposure | 197 | 6 | 1.1 (0.4-3.1) |
| Unexposed | 328 | 9 | 1.0 (ref.) |
| DCE exposure | 198 | 6 | 1.1 (0.4-3.1) |

*during the six months prior to conception

**Additional file 1b.** **Oral clefts and first trimester VOC exposure*, adjusted results, Camp Lejeune, 1968-1985.**

| **Adjusted risk factors** | **Controls**  **#** | **Oral Clefts**  **# OR (95% CI)** | | | |
| --- | --- | --- | --- | --- | --- |
| **Previous pregnancy** |
| Unexposed | 304 | 17 | | 1.0 (ref.) | |
| Low PCE (>0-<44 ppb) | 111 | 4 | | 0.7 (0.2-2.0) | |
| High PCE (≥ 44 ppb) | 111 | 3 | | 0.5 (0.1-1.7) | |
| Unexposed | 253 | 15 | | 1.0 (ref.) | |
| Low TCE (>0-≤2 ppb) | 130 | 4 | | 0.5 (0.2-1.6) | |
| High TCE (> 2 ppb) | 143 | 5 | | 0.6 (0.2-1.8) | |
| Unexposed | 432 | 21 | | 1.0 (ref.) | |
| Benzene exposure | 94 | 3 | | 0.7 (0.2-2.4) | |
| Unexposed | 301 | 17 | | 1.0 (ref.) | |
| Low VC (>0-<3 ppb) | 141 | 4 | | 0.5 (0.2-1.5) | |
| High VC (≥ 3 ppb) | 84 | 3 | | 0.7 (0.2-2.3) | |
| Unexposed | 300 | 17 | | 1.0 (ref.) | |
| Low DCE (>0-<5 ppb) | 116 | 4 | | 0.6 (0.2-2.0) | |
| High DCE (≥ 5 ppb) | 110 | 3 | | 0.5 (0.1-1.7) | |
| **Alcohol use** |  |  | | | |
| Unexposed | 304 | 17 | | 1.0 (ref.) | |
| Low PCE (>0-<44 ppb) | 111 | 4 | | 0.8 (0.2-2.3) | |
| High PCE (≥ 44 ppb) | 111 | 3 | | 0.5 (0.2-1.9) | |
| Unexposed | 253 | 15 | | 1.0 (ref.) | |
| Low TCE (>0-≤2 ppb) | 130 | 4 | | 0.6 (0.2-1.7) | |
| High TCE (> 2 ppb) | 143 | 5 | | 0.5 (0.2-1.6) | |
| Unexposed | 432 | 21 | | 1.0 (ref.) | |
| Benzene exposure | 94 | 3 | | 0.5 (0.1-2.1) | |
| Unexposed | 301 | 17 | | 1.0 (ref.) | |
| Low VC (>0-<3 ppb) | 141 | 4 | | 0.6 (0.2-1.8) | |
| High VC (≥ 3 ppb) | 84 | 3 | | 0.7 (0.2-2.5) | |
| Unexposed | 300 | 17 | | 1.0 (ref.) | |
| Low DCE (>0-<5 ppb) | 116 | 4 | | 0.7 (0.2-2.2) | |
| High DCE (≥ 5 ppb) | 110 | 3 | | 0.5 (0.2-1.9) | |
| **Mother worked** |  |  | | | |
| Unexposed | 304 | 17 | | 1.0 (ref.) | |
| Low PCE (>0-<44 ppb) | 111 | 4 | | 0.7 (0.2-2.3) | |
| High PCE (≥ 44 ppb) | 111 | 3 | | 0.5 (0.1-1.7) | |
| Unexposed | 253 | 15 | | 1.0 (ref.) | |
| Low TCE (>0-≤2 ppb) | 130 | 4 | | 0.6 (0.2-1.8) | |
| High TCE (> 2 ppb) | 143 | 5 | | 0.6 (0.2-1.7) | |
| Unexposed | 432 | 21 | | 1.0 (ref.) | |
| Benzene exposure | 94 | 3 | | 0.7 (0.2-2.4) | |
| Unexposed | 301 | 17 | | 1.0 (ref.) | |
| Low VC (>0-<3 ppb) | 141 | 4 | | 0.5 (0.2-1.7) | |
| High VC (≥ 3 ppb) | 84 | 3 | | 0.7 (0.2-2.3) | |
| Unexposed | 300 | 17 | | 1.0 (ref.) | |
| Low DCE (>0-<5 ppb) | 116 | 4 | | 0.7 (0.2-2.2) | |
| High DCE (≥ 5 ppb) | 110 | 3 | | 0.5 (0.1-1.7) | |
| **Child’s sibling had birth defect** |  |  | | | |
| Unexposed | 304 | 17 | | 1.0 (ref.) | |
| Low PCE (>0-<44 ppb) | 111 | 4 | | 0.8 (0.2-2.4) | |
| High PCE (≥ 44 ppb) | 111 | 3 | | 0.6 (0.2-2.1) | |
| Unexposed | 253 | 15 | | 1.0 (ref.) | |
| Low TCE (>0-≤2 ppb) | 130 | 4 | | 0.6 (0.2-1.9) | |
| High TCE (> 2 ppb) | 143 | 5 | | 0.5 (0.2-1.6) | |
| Unexposed | 432 | 21 | | 1.0 (ref.) | |
| Benzene exposure | 94 | 3 | | 0.5 (0.1-2.2) | |
| Unexposed | 301 | 17 | | 1.0 (ref.) | |
| Low VC (>0-<3 ppb) | 141 | 4 | | 0.6 (0.2-2.0) | |
| High VC (≥ 3 ppb) | 84 | 3 | | 0.8 (0.2-2.7) | |
| Unexposed | 300 | 17 | | 1.0 (ref.) | |
| Low DCE (>0-<5 ppb) | 116 | 4 | | 0.7 (0.2-2.3) | |
| High DCE (≥ 5 ppb) | 110 | 3 | | 0.6 (0.2-2.1) | |
| **Mother’s education** |  |  | | | |
| Unexposed | 304 | 17 | | | 1.0 (ref.) |
| Low PCE (>0-<44 ppb) | 111 | 4 | | | 0.7 (0.2-2.0) |
| High PCE (≥ 44 ppb) | 111 | 3 | | | 0.5 (0.1-1.7) |
| Unexposed | 253 | 15 | | | 1.0 (ref.) |
| Low TCE (>0-≤2 ppb) | 130 | 4 | | | 0.5 (0.2-1.7) |
| High TCE (> 2 ppb) | 143 | 5 | | | 0.6 (0.2-1.8) |
| Unexposed | 432 | 21 | | | 1.0 (ref.) |
| Benzene exposure | 94 | 3 | | | 0.7 (0.2-2.5) |
| Unexposed | 301 | 17 | | | 1.0 (ref.) |
| Low VC (>0-<3 ppb) | 141 | 4 | | | 0.5 (0.2-1.6) |
| High VC (≥ 3 ppb) | 84 | 3 | | | 0.6 (0.2-2.3) |
| Unexposed | 300 | 17 | | | 1.0 (ref.) |
| Low DCE (>0-<5 ppb) | 116 | 4 | | | 0.6 (0.2-1.9) |
| High DCE (≥ 5 ppb) | 110 | 3 | | | 0.5 (0.1-1.7) |
| **Mother used prenatal vitamins** |  |  | | | |
| Unexposed | 304 | 17 | 1.0 (ref.) | | |
| Low PCE (>0-<44 ppb) | 111 | 4 | 0.7 (0.2-2.1) | | |
| High PCE (≥ 44 ppb) | 111 | 3 | 0.5 (0.1-1.8) | | |
| Unexposed | 253 | 15 | 1.0 (ref.) | | |
| Low TCE (>0-≤2 ppb) | 130 | 4 | 0.5 (0.2-1.7) | | |
| High TCE (> 2 ppb) | 143 | 5 | 0.5 (0.2-1.6) | | |
| Unexposed | 432 | 21 | 1.0 (ref.) | | |
| Benzene exposure | 94 | 3 | 0.5 (0.1-2.2) | | |
| Unexposed | 301 | 17 | 1.0 (ref.) | | |
| Low VC (>0-<3 ppb) | 141 | 4 | 0.5 (0.2-1.7) | | |
| High VC (≥ 3 ppb) | 84 | 3 | 0.7 (0.2-2.3) | | |
| Unexposed | 300 | 17 | 1.0 (ref.) | | |
| Low DCE (>0-<5 ppb) | 116 | 4 | 0.6 (0.2-2.0) | | |
| High DCE (≥ 5 ppb) | 110 | 3 | 0.5 (0.1-1.8) | | |
| **Sex of child** |  |  | | | |
| Unexposed | 304 | 17 | | | 1.0 (ref.) |
| Low PCE (>0-<44 ppb) | 111 | 4 | | | 0.6 (0.2-1.9) |
| High PCE (≥ 44 ppb) | 111 | 3 | | | 0.5 (0.1-1.6) |
| Unexposed | 253 | 15 | | | 1.0 (ref.) |
| Low TCE (>0-≤2 ppb) | 130 | 4 | | | 0.5 (0.2-1.6) |
| High TCE (> 2 ppb) | 143 | 5 | | | 0.6 (0.2-1.6) |
| Unexposed | 432 | 21 | | | 1.0 (ref.) |
| Benzene exposure | 94 | 3 | | | 0.7 (0.2-2.2) |
| Unexposed | 301 | 17 | | | 1.0 (ref.) |
| Low VC (>0-<3 ppb) | 141 | 4 | | | 0.5 (0.2-1.5) |
| High VC (≥ 3 ppb) | 84 | 3 | | | 0.6 (0.2-2.1) |
| Unexposed | 300 | 17 | | | 1.0 (ref.) |
| Low DCE (>0-<5 ppb) | 116 | 4 | | | 0.6 (0.2-1.8) |
| High DCE (≥ 5 ppb) | 110 | 3 | | | 0.5 (0.1-1.6) |
| **Maternal fevers** |  |  | | | |
| Unexposed | 304 | 17 | | | 1.0 (ref.) |
| Low PCE (>0-<44 ppb) | 111 | 4 | | | 0.7 (0.2-2.2) |
| High PCE (≥ 44 ppb) | 111 | 3 | | | 0.5 (0.2-1.9) |
| Unexposed | 253 | 15 | | | 1.0 (ref.) |
| Low TCE (>0-≤2 ppb) | 130 | 4 | | | 0.6 (0.2-1.7) |
| High TCE (> 2 ppb) | 143 | 5 | | | 0.5 (0.2-1.7) |
| Unexposed | 432 | 21 | | | 1.0 (ref.) |
| Benzene exposure | 94 | 3 | | | 0.5 (0.1-2.3) |
| Unexposed | 301 | 17 | | | 1.0 (ref.) |
| Low VC (>0-<3 ppb) | 141 | 4 | | | 0.6 (0.2-1.7) |
| High VC (≥ 3 ppb) | 84 | 3 | | | 0.7 (0.2-2.5) |
| Unexposed | 300 | 17 | | | 1.0 (ref.) |
| Low DCE (>0-<5 ppb) | 116 | 4 | | | 0.7 (0.2-2.1) |
| High DCE (≥ 5 ppb) | 110 | 3 | | | 0.5 (0.2-1.9) |

***** when possible, we divided the exposed group by the 50^th^ percentile level among controls (low and high); we excluded categorizations where there were <2 exposed cases in a cell

**Additional file 1c.** **Childhood cancers and first trimester VOC exposure**, adjusted results, Camp Lejeune, 1968-1985.**

| **Adjusted risk factors** | **Controls**  **#** | **Cancers***  **# OR (95% CI)** | |
| --- | --- | --- | --- |
| **Child’s sibling had birth defect** |
| Unexposed | 304 | 6 | 1.0 (ref.) |
| Low PCE (>0-<44 ppb) | 111 | 4 | 1.5 (0.4-6.1) |
| High PCE (≥ 44 ppb) | 111 | 3 | 1.0 (0.2-5.2) |
| Unexposed | 253 | 6 | 1.0 (ref.) |
| Low TCE (>0-≤2 ppb) | 130 | 5 | 1.5 (0.4-5.7) |
| High TCE (> 2 ppb) | 143 | 2 | 0.3 (0.0-2.6) |
| Unexposed | 432 | 11 | 1.0 (ref.) |
| Benzene exposure | 94 | 2 | 1.1 (0.2-5.4) |
| Unexposed | 301 | 6 | 1.0 (ref.) |
| Low VC (>0-<3 ppb) | 141 | 5 | 1.6 (0.4-5.9) |
| High VC (≥ 3 ppb) | 84 | 2 | 0.6 (0.1-5.4) |
| Unexposed | 300 | 6 | 1.0 (ref.) |
| Low DCE (>0-<5 ppb) | 116 | 4 | 1.4 (0.3-5.8) |
| High DCE (≥ 5 ppb) | 110 | 3 | 1.0 (0.2-5.2) |
| **Mother’s education** |  |  | |
| Unexposed | 304 | 6 | 1.0 (ref.) |
| Low PCE (>0-<44 ppb) | 111 | 4 | 1.7 (0.5-6.2) |
| High PCE (≥ 44 ppb) | 111 | 3 | 1.2 (0.3-5.0) |
| Unexposed | 253 | 6 | 1.0 (ref.) |
| Low TCE (>0-≤2 ppb) | 130 | 5 | 1.5 (0.5-5.1) |
| High TCE (> 2 ppb) | 143 | 2 | 0.6 (0.1-2.9) |
| Unexposed | 432 | 11 | 1.0 (ref.) |
| Benzene exposure | 94 | 2 | 0.9 (0.2-4.1) |
| Unexposed | 301 | 6 | 1.0 (ref.) |
| Low VC (>0-<3 ppb) | 141 | 5 | 1.6 (0.5-5.5) |
| High VC (≥ 3 ppb) | 84 | 2 | 1.1 (0.1-5.5) |
| Unexposed | 300 | 6 | 1.0 (ref.) |
| Low DCE (>0-<5 ppb) | 116 | 4 | 1.6 (0.4-5.9) |
| High DCE (≥ 5 ppb) | 110 | 3 | 1.2 (0.3-5.0) |
| **Maternal smoking** |  |  | |
| Unexposed | 304 | 6 | 1.0 (ref.) |
| Low PCE (>0-<44 ppb) | 111 | 4 | 1.8 (0.5-6.6) |
| High PCE (≥ 44 ppb) | 111 | 3 | 0.9 (0.2-4.4) |
| Unexposed | 253 | 6 | 1.0 (ref.) |
| Low TCE (>0-≤2 ppb) | 130 | 5 | 1.6 (0.5-5.3) |
| High TCE (> 2 ppb) | 143 | 2 | 0.3 (0.0-2.6) |
| Unexposed | 432 | 11 | 1.0 (ref.) |
| Benzene exposure | 94 | 2 | 1.0 (0.2-4.6) |
| Unexposed | 301 | 6 | 1.0 (ref.) |
| Low VC (>0-<3 ppb) | 141 | 5 | 1.7 (0.5-5.8) |
| High VC (≥ 3 ppb) | 84 | 2 | 0.6 (0.1-4.9) |
| Unexposed | 300 | 6 | 1.0 (ref.) |
| Low DCE (>0-<5 ppb) | 116 | 4 | 1.7 (0.5-6.1) |
| High DCE (≥ 5 ppb) | 110 | 3 | 0.9 (0.2-4.5) |
| **Maternal fever** |  |  | |
| Unexposed | 304 | 6 | 1.0 (ref.) |
| Low PCE (>0-<44 ppb) | 111 | 4 | 2.1 (0.6-8.1) |
| High PCE (≥ 44 ppb) | 111 | 3 | 1.1 (0.2-5.6) |
| Unexposed | 253 | 6 | 1.0 (ref.) |
| Low TCE (>0-≤2 ppb) | 130 | 5 | 2.0 (0.6-7.0) |
| High TCE (> 2 ppb) | 143 | 2 | 0.4 (0.0-3.6) |
| Unexposed | 432 | 11 | 1.0 (ref.) |
| Benzene exposure | 94 | 2 | 1.2 (0.3-5.7) |
| Unexposed | 301 | 6 | 1.0 (ref.) |
| Low VC (>0-<3 ppb) | 141 | 5 | 2.1 (0.6-7.6) |
| High VC (≥ 3 ppb) | 84 | 2 | 0.7 (0.1-6.2) |
| Unexposed | 300 | 6 | 1.0 (ref.) |
| Low DCE (>0-<5 ppb) | 116 | 4 | 2.0 (0.5-7.7) |
| High DCE (≥ 5 ppb) | 110 | 3 | 1.1 (0.2-5.9) |
| **Passive smoking** |  |  | |
| Unexposed | 304 | 6 | 1.0 (ref.) |
| Low PCE (>0-<44 ppb) | 111 | 4 | 1.7 (0.5-6.2) |
| High PCE (≥ 44 ppb) | 111 | 3 | 0.8 (0.2-4.3) |
| Unexposed | 253 | 6 | 1.0 (ref.) |
| Low TCE (>0-≤2 ppb) | 130 | 5 | 1.5 (0.5-5.1) |
| High TCE (> 2 ppb) | 143 | 2 | 0.3 (0.0-2.5) |
| Unexposed | 432 | 11 | 1.0 (ref.) |
| Benzene exposure | 94 | 2 | 1.0 (0.2-4.6) |
| Unexposed | 301 | 6 | 1.0 (ref.) |
| Low VC (>0-<3 ppb) | 141 | 5 | 1.7 (0.5-5.6) |
| High VC (≥ 3 ppb) | 84 | 2 | 0.6 (0.1-4.9) |
| Unexposed | 300 | 6 | 1.0 (ref.) |
| Low DCE (>0-<5 ppb) | 116 | 4 | 1.6 (0.4-5.7) |
| High DCE (≥ 5 ppb) | 110 | 3 | 0.9 (0.2-4.4) |
| **Mother’s race** |  |  | |
| Unexposed | 304 | 6 | 1.0 (ref.) |
| Low PCE (>0-<44 ppb) | 111 | 4 | 1.7 (0.5-6.3) |
| High PCE (≥ 44 ppb) | 111 | 3 | 1.2 (0.3-5.1) |
| Unexposed | 253 | 6 | 1.0 (ref.) |
| Low TCE (>0-≤2 ppb) | 130 | 5 | 1.6 (0.5-5.2) |
| High TCE (> 2 ppb) | 143 | 2 | 0.6 (0.1-2.8) |
| Unexposed | 432 | 11 | 1.0 (ref.) |
| Benzene exposure | 94 | 2 | 0.9 (0.2-4.0) |
| Unexposed | 301 | 6 | 1.0 (ref.) |
| Low VC (>0-<3 ppb) | 141 | 5 | 1.7 (0.5-5.6) |
| High VC (≥ 3 ppb) | 84 | 2 | 1.1 (0.2-5.5) |
| Unexposed | 300 | 6 | 1.0 (ref.) |
| Low DCE (>0-<5 ppb) | 116 | 4 | 1.7 (0.5-6.0) |
| High DCE (≥ 5 ppb) | 110 | 3 | 1.2 (0.3-5.0) |
| **Father smoked** |  |  | |
| Unexposed | 299 | 6 | 1.0 (ref.) |
| Low PCE (>0-<44 ppb) | 109 | 4 | 1.8 (0.5-6.5) |
| High PCE (≥ 44 ppb) | 111 | 3 | 1.3 (0.3-5.5) |
| Unexposed | 248 | 6 | 1.0 (ref.) |
| Low TCE (>0-≤2 ppb) | 128 | 5 | 1.5 (0.5-5.1) |
| High TCE (> 2 ppb) | 143 | 2 | 0.6 (0.1-2.9) |
| Unexposed | 426 | 11 | 1.0 (ref.) |
| Benzene exposure | 93 | 2 | 0.8 (0.2-3.8) |
| Unexposed | 296 | 6 | 1.0 (ref.) |
| Low VC (>0-<3 ppb) | 139 | 5 | 1.7 (0.5-5.7) |
| High VC (≥ 3 ppb) | 84 | 2 | 1.2 (0.2-6.2) |
| Unexposed | 295 | 6 | 1.0 (ref.) |
| Low DCE (>0-<5 ppb) | 114 | 4 | 1.6 (0.5-6.0) |
| High DCE (≥ 5 ppb) | 110 | 3 | 1.4 (0.3-5.6) |
| **Sex of child** |  |  | |
| Unexposed | 304 | 6 | 1.0 (ref.) |
| Low PCE (>0-<44 ppb) | 111 | 4 | 1.9 (0.5-6.7) |
| High PCE (≥ 44 ppb) | 111 | 3 | 1.4 (0.3-5.7) |
| Unexposed | 253 | 6 | 1.0 (ref.) |
| Low TCE (>0-≤2 ppb) | 130 | 5 | 1.6 (0.5-5.5) |
| High TCE (> 2 ppb) | 143 | 2 | 0.6 (0.1-3.0) |
| Unexposed | 432 | 11 | 1.0 (ref.) |
| Benzene exposure | 94 | 2 | 0.8 (0.2-3.8) |
| Unexposed | 301 | 6 | 1.0 (ref.) |
| Low VC (>0-<3 ppb) | 141 | 5 | 1.8 (0.5-6.0) |
| High VC (≥ 3 ppb) | 84 | 2 | 1.2 (0.2-6.2) |
| Unexposed | 300 | 6 | 1.0 (ref.) |
| Low DCE (>0-<5 ppb) | 116 | 4 | 1.7 (0.5-6.3) |
| High DCE (≥ 5 ppb) | 110 | 3 | 1.4 (0.3-5.8) |
| **Dad possibly exposed to Agent Orange** |  |  | |
| Unexposed | 291 | 6 | 1.0 (ref.) |
| Low PCE (>0-<44 ppb) | 110 | 4 | 1.7 (0.5-6.3) |
| High PCE (≥ 44 ppb) | 105 | 3 | 1.4 (0.3-5.8) |
| Unexposed | 242 | 6 | 1.0 (ref.) |
| Low TCE (>0-≤2 ppb) | 127 | 5 | 1.5 (0.4-5.2) |
| High TCE (> 2 ppb) | 137 | 2 | 0.6 (0.1-3.0) |
| Unexposed | 414 | 11 | 1.0 (ref.) |
| Benzene exposure | 92 | 2 | 0.8 (0.2-3.6) |
| Unexposed | 289 | 6 | 1.0 (ref.) |
| Low VC (>0-<3 ppb) | 138 | 5 | 1.7 (0.5-5.7) |
| High VC (≥ 3 ppb) | 79 | 2 | 1.3 (0.3-7.2) |
| Unexposed | 288 | 6 | 1.0 (ref.) |
| Low DCE (>0-<5 ppb) | 114 | 4 | 1.6 (0.5-5.9) |
| High DCE (≥ 5 ppb) | 104 | 3 | 1.5 (0.4-6.0) |

*childhood leukemia and childhood non-Hodgkin’s lymphoma

****** when possible, we divided the exposed group by the 50^th^ percentile level among controls (low and high); we excluded categorizations where there were <2 exposed cases in a cell
