# Supplementary material for: Evaluation of exposure to contaminated drinking water and specific birth defects and childhood cancers at Marine Corps Base Camp Lejeune, North Carolina: a case–control study
Source: Environ Health. 2013 Dec 4;12:104. doi: 10.1186/1476-069X-12-104 (PMC3880212; doi:10.1186/1476-069X-12-104)
Supplement: Additional file 2 — Neural tube defects and first trimester VOC exposure (unexposed group had no exposure to any VOCs), Camp Lejeune, 1968-1985. [file 1476-069X-12-104-S2.doc]

**Additional file 2. Neural tube defects and first trimester VOC exposure (unexposed group had no exposure to any VOCs), Camp Lejeune, 1968-1985.**

|  | **Controls**  **#** | **Neural Tube Defects**  **# OR (95% CI)** | |
| --- | --- | --- | --- |
| **PCE** |  |  | |
| Unexposed to any VOCs | 287 | 7 | 1.0 (ref.) |
| PCE > 5 ppb | 169 | 2 | 0.5 (0.1-2.4) |
| Unexposed to any VOCs | 287 | 7 | 1.0 (ref.) |
| PCE > 0 ppb | 196 | 5 | 1.0 (0.3-3.3) |
| **Benzene** |  |  | |
| Unexposed to any VOCs | 287 | 7 | 1.0 (ref.) |
| Benzene > 0 ppb | 73 | 6 | 3.4 (1.1-10.3) |
| **Vinyl Chloride** |  |  | |
| Unexposed to any VOCs | 287 | 7 | 1.0 (ref.) |
| VC > 0 ppb | 197 | 6 | 1.2 (0.4-3.8) |
| **DCE** |  |  | |
| Unexposed to any VOCs | 297 | 7 | 1.0 (ref.) |
| DCE > 0 ppb | 198 | 6 | 1.2 (0.4-3.8) |

| **Note: TCE tables with group unexposed to any VOCs are the same as TCE tables comparing TCE exposure with no exposure to TCE because there are no instances where a person would have PCE but no TCE** |
| --- |
