# Supplementary material for: Evaluation of exposure to contaminated drinking water and specific birth defects and childhood cancers at Marine Corps Base Camp Lejeune, North Carolina: a case–control study
Source: Environ Health. 2013 Dec 4;12:104. doi: 10.1186/1476-069X-12-104 (PMC3880212; doi:10.1186/1476-069X-12-104)
Supplement: Additional file 3 — Oral clefts and childhood cancers and first trimester VOC exposure (unexposed group had no exposure to any VOCs), Camp Lejeune, 1968-1985. [file 1476-069X-12-104-S3.doc]

**Additional file 3. Oral clefts and childhood cancers and first trimester VOC exposure (unexposed group had no exposure to any VOCs), Camp Lejeune, 1968-1985.**

|  | **Controls**  **#** | **Oral Clefts**  **# OR (95% CI)** | | **Cancers***  **# OR (95% CI)** | |
| --- | --- | --- | --- | --- | --- |
| **PCE** |  |  |  |  | |
| Unexposed to any VOCs | 253 | 15 | 1.0 (ref.) | 6 | 1.0 (ref.) |
| >0-<44 ppb | 111 | 4 | 0.6 (0.2-1.9) | 4 | 1.5 (0.4-5.5) |
| ≥ 44 ppb | 111 | 3 | 0.5 (0.1-1.6) | 3 | 1.1 (0.3-4.6) |
| Unexposed to any VOCs | 253 | 15 | 1.0 (ref.) | 6 | 1.0 (ref.) |
| PCE > 5 ppb | 185 | 5 | 0.5 (0.2-1.3) | 6 | 1.4 (0.4-4.3) |
| Unexposed to any VOCs | 253 | 15 | 1.0 (ref.) | 6 | 1.0 (ref.) |
| PCE > 0 ppb | 222 | 7 | 0.5 (0.2-1.3) | 7 | 1.3 (0.4-4.0) |
| **Benzene** |  |  | |  | |
| Unexposed to any VOCs | 251 | 15 | 1.0 (ref.) | 6 | 1.0 (ref.) |
| Benzene > 0 ppb | 94 | 3 | 0.5 (0.2-1.9) | 2 | 0.9 (0.2-4.5) |
| **Vinyl Chloride** |  |  | |  | |
| Unexposed to any VOCs | 253 | 15 | 1.0 (ref.) | 6 | 1.0 (ref.) |
| >0-<3 ppb | 141 | 4 | 0.5 (0.2-1.5) | 5 | 1.5 (0.4-5.0) |
| ≥ 3 ppb | 84 | 3 | 0.6 (0.2-2.1) | 2 | 1.0 (0.2-5.1) |
| Unexposed | 253 | 15 | 1.0 (ref.) | 6 | 1.0 (ref.) |
| <0-≤2 pbb | 74 | 4 | 0.9 (0.3-2.8) | 3 | 1.7 (0.4-7.0) |
| > 2 ppb | 151 | 3 | 0.3 (0.1-1.2) | 4 | 1.1 (0.3-4.0) |
| Unexposed to any VOCs | 253 | 15 | 1.0 (ref.) | 6 | 1.0 (ref.) |
| VC > 0 ppb | 225 | 7 | - 1. (0.2-1.3) | 7 | 1.3 (0.4-4.0) |
| **DCE** |  |  | |  | |
| Unexposed | 253 | 15 | 1.0 (ref.) | 6 | 1.0 (ref.) |
| >0-<5 ppb | 116 | 4 | 0.6 (0.2-1.8) | 4 | 1.5 (0.4-5.3) |
| ≥ 5 ppb | 110 | 3 | 0.5 (0.1-1.6) | 3 | 1.2 (0.3-4.7) |
| Unexposed to any VOCs | 253 | 15 | 1.0 (ref.) | 6 | 1.0 (ref.) |
| DCE > 0 ppb | 226 | 7 | 0.5 (0.2-1.3) | 7 | 1.3 (0.4-3.9) |

*childhood leukemia and childhood non-Hodgkin’s lymphoma
