# Supplementary material for: Evaluation of exposure to contaminated drinking water and specific birth defects and childhood cancers at Marine Corps Base Camp Lejeune, North Carolina: a case–control study
Source: Environ Health. 2013 Dec 4;12:104. doi: 10.1186/1476-069X-12-104 (PMC3880212; doi:10.1186/1476-069X-12-104)
Supplement: Additional file 4 — Neural tube defects and first trimester VOC exposure, accounting for water consumption, Camp Lejeune, 1968-1985*. [file 1476-069X-12-104-S4.doc]

**Additional file 4. Neural tube defects and first trimester VOC exposure, accounting for water consumption, Camp Lejeune, 1968-1985.***

|  | **Controls**  **#** | **Neural Tube Defects**  **# OR (95% CI)** | |
| --- | --- | --- | --- |
| **TCE** |  |  | |
| Unexposed | 287 | 7 | 1.0 (ref.) |
| Exposed (≤ 5 glasses/day) | 72 | 2 | 1.1 (0.2-5.6) |
| Exposed > 5 glasses/day) | 120 | 6 | 2.1 (0.7-6.2) |
| **VC** |  |  | |
| Unexposed | 329 | 9 | 1.0 (ref.) |
| Exposed (≤ 5 glasses/day) | 47 | 2 | 1.6 (0.3-7.4) |
| Exposed > 5 glasses/day) | 112 | 4 | 1.3 (0.4-4.3) |
| **DCE** |  |  | |
| Unexposed | 328 | 9 | 1.0 (ref.) |
| Exposed (≤ 5 glasses/day) | 48 | 2 | 1.5 (0.3-7.2) |
| Exposed > 5 glasses/day) | 112 | 4 | 1.3 (0.4-4.3) |

* less than two exposed cases in one of the cells for PCE and benzene exposure
