# Supplementary material for: Evaluation of exposure to contaminated drinking water and specific birth defects and childhood cancers at Marine Corps Base Camp Lejeune, North Carolina: a case–control study
Source: Environ Health. 2013 Dec 4;12:104. doi: 10.1186/1476-069X-12-104 (PMC3880212; doi:10.1186/1476-069X-12-104)
Supplement: Additional file 5 — Oral clefts and childhood cancers and first trimester VOC exposure, accounting for water consumption, Camp Lejeune, 1968-1985*. [file 1476-069X-12-104-S5.doc]

**Additional file 5. Oral clefts and childhood cancers and first trimester VOC exposure, accounting for water consumption, Camp Lejeune, 1968-1985.***

|  | **Controls**  **#** | **Oral Clefts**  **# OR (95% CI)** | |
| --- | --- | --- | --- |
| **TCE** |  |  | |
| Unexposed | 253 | 15 | 1.0 (ref.) |
| Exposed (≤ 5 glasses/day) | 83 | 2 | 0.4 (0.1-1.8) |
| Exposed > 5 glasses/day) | 139 | 6 | 0.7 (0.3-1.9) |

* less than two exposed cases in one of the cells for PCE, benzene, VC, and DCE exposure for oral clefts and less than two exposed cases in one of the cells for any contaminant and childhood cancers
